# Omega-3 Fatty Acid Intervention Suppresses Lipopolysaccharide-Induced Inflammation and Weight Loss in Mice

**DOI:** 10.3390/md13021026

**Published:** 2015-02-13

**Authors:** Ying-Hua Liu, Xiang-Yong Li, Chih-Yu Chen, Hong-Man Zhang, Jing X. Kang

**Affiliations:** 1 Laboratory for Lipid Medicine and Technology (LLMT), Massachusetts General Hospital and Harvard Medical School, Boston, MA 02129, USA; E-Mails: liuyinghua77@hotmail.com (Y.-H.L.); xyli75@126.com (X.-Y.L.); cchen45@mgh.harvard.edu (C.-Y.C.); hmzhang9607@sina.com (H.-M.Z.); 2 Nutrition Department, People’s Liberation Army General Hospital, Beijing 100853, China; 3 Institute of Biochemistry and Molecular Biology, Guangdong Medical College, Zhanjiang, Guangdong 524023, China

**Keywords:** *n*-3 polyunsaturated fatty acids, lipopolysaccharide, inflammation, muscle atrophy, TLR4, sepsis

## Abstract

Bacterial endotoxin lipopolysaccharide (LPS)-induced sepsis is a critical medical condition, characterized by a severe systemic inflammation and rapid loss of muscle mass. Preventive and therapeutic strategies for this complex disease are still lacking. Here, we evaluated the effect of omega-3 (*n*-3) polyunsaturated fatty acid (PUFA) intervention on LPS-challenged mice with respect to inflammation, body weight and the expression of Toll-like receptor 4 (TLR4) pathway components. LPS administration induced a dramatic loss of body weight within two days. Treatment with *n*-3 PUFA not only stopped loss of body weight but also gradually reversed it back to baseline levels within one week. Accordingly, the animals treated with *n*-3 PUFA exhibited markedly lower levels of inflammatory cytokines or markers in plasma and tissues, as well as down-regulation of TLR4 pathway components compared to animals without *n*-3 PUFA treatment or those treated with omega-6 PUFA. Our data demonstrate that *n*-3 PUFA intervention can suppress LPS-induced inflammation and weight loss via, at least in part, down-regulation of pro-inflammatory targets of the TLR4 signaling pathway, and highlight the therapeutic potential of *n*-3 PUFA in the management of sepsis.

## 1. Introduction

Sepsis is a potentially life-threatening complication of an infection, characterized by a systemic inflammatory response syndrome and rapid loss of muscle mass and myofibrillar protein content, or muscle atrophy [1,2]. Lipopolysaccharide (LPS), an endotoxin located on the outer cell membrane of Gram-negative bacteria, is a major factor triggering septic shock and inflammatory response [3]. The global incidence of sepsis and septic shock has increased over the past two decades and is predicted to continue to rise over the next 20 years [1]. Identification of safe and effective means for the management of sepsis is warranted. Nutritional modulation aimed at suppressing the release of pro-inflammatory cytokines and preventing weight loss may be of potential benefit in attenuating muscle atrophy and inflammation during sepsis.

Accumulating evidence indicates that omega-3 (*n*-3) polyunsaturated fatty acids (PUFA) such as eicosapentaenoic acid (EPA, 20:5*n*-3) and docosahexaenoic acid (DHA, 22:6*n*-3) play beneficial roles in the management of inflammatory diseases [4,5,6]. Omega-3 PUFA have also been shown to improve muscle protein synthesis [7,8], and alleviate muscle atrophy in cancer [9] and sepsis [10]. Collectively, these data suggest that *n*-3 PUFA represent potentially novel therapeutic agents for critically ill and severely injured patients who commonly present with inflammation and weight loss problems. The majority of studies in this area, however, have focused on the preventive effects of fish oil (FO) through dietary supplementation [11,12]. Whether FO intervention is effective in suppressing LPS-induced inflammation and weight loss requires further investigation. Moreover, although the beneficial role of *n*-3 PUFA is thought to be associated with their inhibitory effects on overproduction of pro-inflammatory cytokines [4,5,6], the specific signaling pathways are unclear.

Toll-like receptors (TLRs) play critical roles in the induction of inflammatory and immune responses against conserved microbial structures, referred to as pathogen-associated molecular patterns (PAMPs) [13]. One such PAMP is LPS. TLR4 is a subclass of the TLR family involved in the activation of the innate immune and inflammatory response in mammals upon the binding of LPS [14,15]. The intestine is rich in TLR4 receptors and is often the first organ to respond to increased LPS generated by intestinal bacteria. The interaction of TLR4 subclass members with LPS and other PAMPs engages intracellular signaling pathways that culminate in the activation of nuclear factor κB (NFκB) and the induction of inflammatory genes encoding tumor necrosis factor-α (TNF-α), interleukin-1β (IL-1β), and IL-6 [16]. Moreover, inhibition by unsaturated fatty acids of LPS-induced NFκB activation has been attributed to suppression of TLR4-derived signaling pathways [17]. In this context, this study was designed to test the hypothesis that intervention with *n*-3 PUFA-containing FO protects against LPS-induced weight loss and inflammatory cytokine production by suppressing the TLR4 signaling pathway.

## 2. Results

### 2.1. Effect of FO Intervention on LPS-Induced Weight Loss

**Figure 1:**
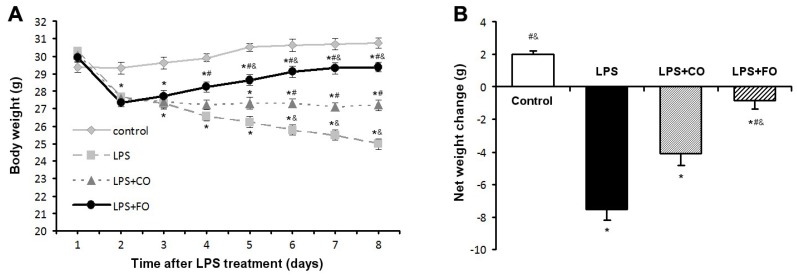
Effect of fish oil (FO) intervention on body weight in LPS-challenged mice. Animals were challenged with LPS on Day 1, with FO and CO intervention on Day 2. (**A**) Body weight measurements taken over the study duration; (**B**) Net body weight change. Values are means ± SE (*n* = 6). * *p* < 0.05 compared to the control group, # *p* < 0.05 compared to the LPS group, & *p* < 0.05 compared to the LPS plus corn oil (LPS + CO) group.

Following the challenge with LPS (3.5 mg/kg intraperitoneal injection), body weight (BW) and food intake were monitored daily. During the study period, total food intake did not differ significantly among the four groups (Table 1). As anticipated, compared with the control group without LPS challenge, body weight on Day 2 after LPS administration was significantly reduced in all LPS-treated animals (LPS, LPS + CO, and LPS + FO) (Figure 1A). Interestingly, supplementation of the LPS-challenged mice with fish oil containing 63% EPA and DHA (by gavage of 100 µL·FO/mouse/day) stopped BW drop and even raised it gradually, resulting in a significant difference in BW on Day 8 between the animals treated with FO (LPS + FO) and those without supplementation (LPS) or with corn oil (CO, containing 62% *n*-6 PUFA) supplementation (LPS + CO) (Figure 1A). Furthermore, analysis of the net BW change over the course of the study (Figure 1B) indicated that FO intervention opposed LPS-induced weight loss in the mice.

**Table 1:** Weight of food intake per day (g) of mice during the study (*n* = 6).

Group	1 day	2 days	3 days	4 days	5 days	6 days	7 days	8 days
Control	3.38 ± 0.08	3.20 ± 0.09	3.29 ± 0.08	3.19 ± 0.09	3.24 ± 0.16	3.09 ± 0.08	2.97 ± 0.08	3.15 ± 0.11
LPS	3.37 ± 0.14	3.33 ± 0.11	3.21 ± 0.13	3.19 ± 0.08	3.00 ± 0.14	2.98 ± 0.06	2.96 ± 0.10	3.11 ± 0.13
LPS + CO	3.42 ± 0.09	3.23 ± 0.12	3.24 ± 0.09	3.06 ± 0.09	3.19 ± 0.14	3.02 ± 0.10	2.94 ± 0.12	3.08 ± 0.12
LPS + FO	3.30 ± 0.10	3.17 ± 0.09	3.23 ± 0.10	3.11 ± 0.11	3.13 ± 0.12	2.98 ± 0.12	2.97 ± 0.08	3.14 ± 0.09
*F*	0.7475	1.447	0.3475	1.228	3.2200	0.8075	0.0600	0.3000
*P*	0.5536	0.2997	0.7922	0.3613	0.0826	0.5243	0.9794	0.8247

CO, corn oil; FO, fish oil. LPS + CO and LPS + FO groups received additional calories per day by gavage (~6% of total energy intake).

### 2.2. Effect of FO Intervention on LPS-Induced Inflammation

**Figure 2:**
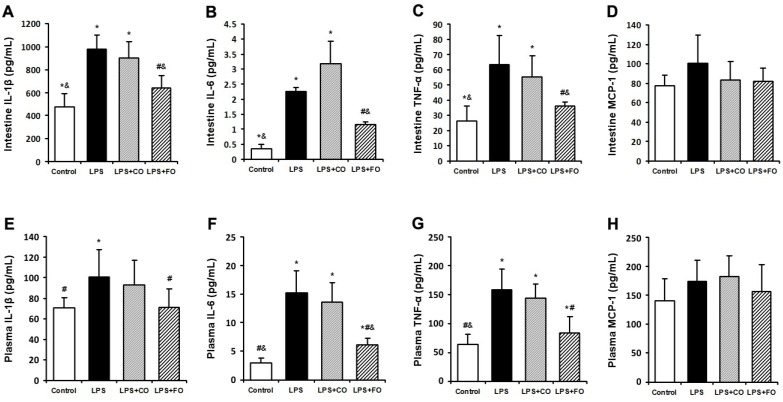
Effect of fish oil (FO) intervention on cytokine levels in lipopolysaccharide (LPS)-challenged mice. Small intestine tissue levels of (**A**) IL-1β; (**B**) IL-6; (**C**) TNF-α; and (**D**) MCP-1. Plasma levels of (**E**) IL-1β; (**F**) IL-6; (**G**) TNF-α; and (**H**) MCP-1. Values are means ± SE (*n* = 6). * *p* < 0.05 compared to the control group, # *p* < 0.05 compared to the LPS group, & *p* < 0.05 compared to the LPS + CO group.

**Figure 3:**
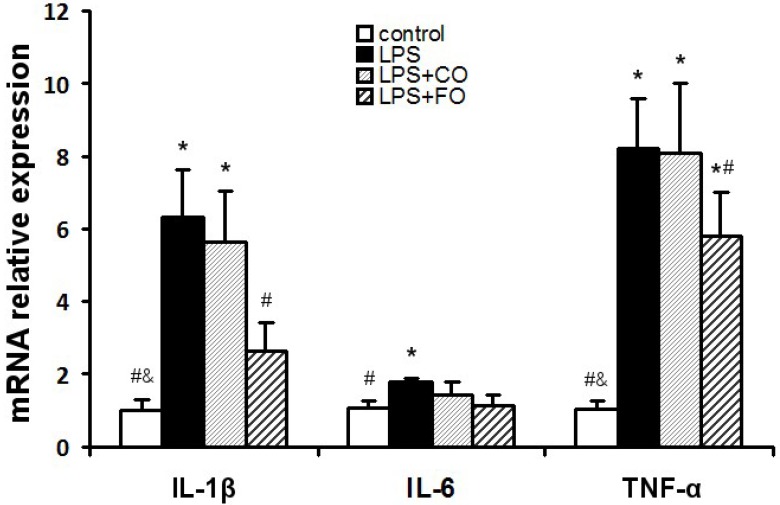
Effect of FO intervention on small intestine mRNA levels of cytokine genes in LPS-challenged mice. Values are means ± SE (*n* = 6). * *p* < 0.05 compared to the control group, # *p* < 0.05 compared to the LPS group, & *p* < 0.05 compared to the LPS + CO group.

**Figure 4:**
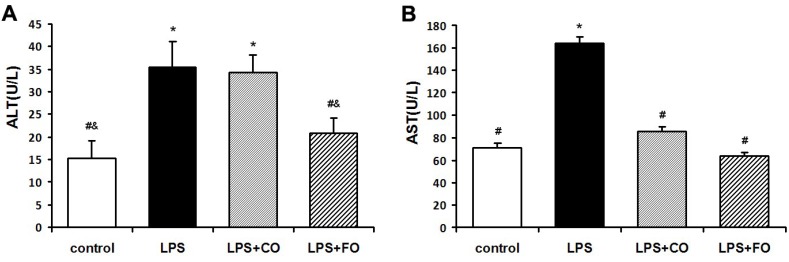
Effect of FO intervention on plasma ALT (**A**) and AST (**B**) activities in LPS-challenged mice. Values are means ± SE (*n* = 6). * *p* < 0.05 compared to the control group, # *p* < 0.05 compared to the LPS group, & *p* < 0.05 compared to the LPS + CO group.

We next examined the effect of FO on LPS-induced changes in the levels of inflammation-related cytokines. As LPS was administered by intraperitoneal injection, and may readily interact with the abundant TLR4 receptors in the intestine, we measured changes in inflammatory status in the plasma and intestine. LPS challenge induced a dramatic increase in the levels of IL-1β, IL-6, and TNF-α in both plasma and small intestine (Figure 2), as measured by multiplex immunoassay. FO intervention markedly reduced the small intestine and plasma levels of the inflammatory cytokines, whereas CO supplementation failed to decrease the cytokine levels significantly, as the cytokine levels in the LPS + FO group, but not in the LPS + CO group, were significantly lower than those of the LPS group (Figure 2). In addition, examination of mRNA expression levels of TNF-α, IL-1β, and IL-6 in the small intestine tissue showed a similar suppressing effect by FO intervention (Figure 3). Furthermore, we measured plasma ALT and AST activities as markers of liver inflammation. As shown in Figure 4, LPS challenge resulted in increased plasma levels of both ALT and AST, which were significantly reduced by FO intervention, suggesting that omega-3 PUFA protect against LPS-induced liver inflammation and damage.

### 2.3. Effect of FO Intervention on the Expression of TLR4 Pathway Components

**Figure 5:**
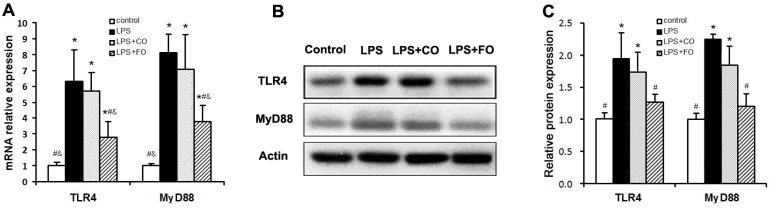
Effect of FO intervention on mRNA (**A**) and protein (B-C) expression of TLR4 and myD88 in small intestine of LPS-challenged mice. Values are means ± SE (*n* = 6 for mRNA expression, *n* = 3 for protein expression). (**B**) Western blot images; (**C**) Gray-scale analysis of Western blot images. * *p* < 0.05 compared to the control group, # *p* < 0.05 compared to the LPS group, & *p* < 0.05 compared to the LPS + CO group.

**Figure 6:**
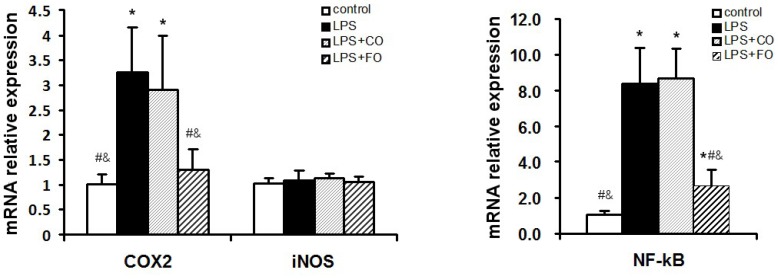
Effect of FO intervention on small intestine mRNA levels of TLR4 signaling pathway factors in LPS-challenged mice. Values are means ± SE (*n* = 6). * *p* < 0.05 compared to the control group, # *p* < 0.05 compared to the LPS group, & *p* < 0.05 compared to the LPS + CO group.

**Figure 7:**
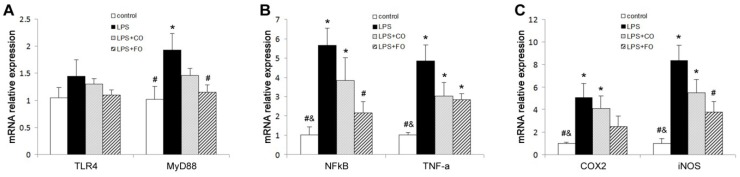
Effect of FO intervention on muscle mRNA levels of TLR4 signaling pathway factors in LPS-challenged mice. mRNA relative expression of (**A**) TLR4 and MyD88; (**B**) NFκB and TNF-α; (**C**) COX-2 and iNOS. Values are means ± SE (*n* = 6). * *p* < 0.05 compared to the control group, # *p* < 0.05 compared to the LPS group, & *p* < 0.05 compared to the LPS + CO group.

We proceeded to evaluate the effects of FO intervention on LPS-induced changes in the levels of components of the TLR4 signaling pathway. LPS challenge remarkably upregulated small intestine expression of TLR4 and MyD88, as shown by increased levels of both mRNA and protein of these factors (Figure 5). Interestingly, FO intervention, but not CO intervention, was able to significantly suppress the LPS-induced upregulation of the genes (Figure 5). Furthermore, we determined the mRNA levels of downstream components of the TLR4 pathway, such as NFκB, COX2, and iNOS, in the small intestine. As shown in Figure 6, the increased expression of NFκB and COX2 induced by LPS challenge could be significantly suppressed by FO intervention, but not CO intervention. Similar results on the differential effects of FO and CO interventions on the expression of the pathway components were found in skeletal muscle (Figure 7). These data indicate that FO intervention can down-regulate pro-inflammatory targets of the TLR4 signaling pathway.

## 3. Discussion

Septic shock encompasses a variety of systemic and cellular processes that collectively lead to overproduction of inflammatory mediators and culminate in weight loss, organ failure, and death. The Gram-negative bacterial endotoxin LPS is a potent activator of the immune system, inducing the production of inflammatory mediators, such as TNF-α, IL-1β and IL-6, which have been implicated in the early phase of shock [1,3]. In this study, we used i.p. injection of LPS to induce septic conditions and to test the interventional effects of *n*-3 PUFA. Our data demonstrated that *n*-3 PUFA FO can significantly reduce weight loss and inflammatory status, the two major manifestations of sepsis.

Omega-3 fatty acids, such as EPA and DHA mainly found in FO, have been shown to decrease the production of inflammatory cytokines and exert beneficial effects on inflammation-related diseases in animal models and clinical trials [4,5,6]. Consistent with these observations, we found that FO intervention resulted in decreased tissue levels of TNF-α, IL-1β, and IL-6 and decreased plasma ALT and AST activities, indicating reduced systemic and hepatic inflammation. In this study, we used corn oil (containing 60% *n*-6 PUFA) as an isoenergetic control for the FO group, and found that CO supplementation did not result in similar benefits, suggesting a differential effect of *n*-6 and *n*-3 PUFA on septic conditions. It is notable that the CO intervention group did exhibit some beneficial effects against the decline in body weight, but not inflammatory status. This may be due to the additional energy intake from fat provided by CO intervention, compared to the LPS group.

TLR4 is involved in the recognition of LPS in the outer membrane of Gram-negative bacteria and helps to trigger the subsequent inflammatory response [18,19]. When bound by LPS, the TLR4/CD14/LBP receptor complex engages MyD88 to trigger a downstream signaling cascade that results in activation of NFκB and the inductions of genes encoding pro-inflammatory cytokines and COX2 [18]. In this context, we designed our study to examine whether FO intervention affects factors involved in the LPS-induced TLR4 signaling pathway. Given that the main physiological effect of LPS challenge was weight loss, secondary to intestine and muscle atrophy, we focused on evaluating the expression levels of TLR4 signaling pathway components and transcriptional targets in intestine and skeletal muscle. We found that FO intervention suppressed the LPS-induced upregulation of TLR4, MyD88, NFκB, TNF-α, IL-1β, and COX2 in both tissues. These findings suggest that the beneficial effects of FO intervention on LPS-induced weight loss and inflammation may be associated with the suppression of TLR4 signaling. A limitation of this study is the lack of data on body composition among the groups, which would provide further insight into the effects of FO intervention.

To date, few *in vivo* studies have examined the role of *n*-3 PUFA in the modulation of TLR signaling [20,21,22]. One such study by Liu *et al.* [20] suggested that fish oil may suppress muscle pro-inflammatory cytokine production in weanling piglets via regulation of TLR and NOD signaling pathways. Nevertheless, several *in vitro* studies have reported that *n*-3 EPA and DHA inhibit the activation of TLR4 and the expression of its target genes [23]. Moreover, *n*-3 PUFA have been shown to inhibit LPS or lauric acid-induced activation of TLR4 through inhibition of receptor dimerization and recruitment into lipid rafts in a reactive oxygen species-dependent manner [24]. Our findings that FO intervention in mice suppresses LPS-induced expression of inflammatory cytokines and factors in the TLR4 signaling pathway support the notion that modulation of TLR4 pathway signaling is a key anti-inflammatory mechanism of *n*-3 PUFA.

## 4. Materials and Methods

### 4.1. Animals and Treatment

Nine-week old male C57BL/6 mice (*n* = 24) were purchased from Charles River Laboratories (Boston, MA, USA). All mice were housed in metal barred cages (3 mice/cage), had 12-h light and dark cycles, and were fed a regular chow diet. After arrival, mice went through a 7-day acclimatization period, after which six mice were designated as the control group and injected with 0.1 mL PBS, while the remaining 18 mice were challenged with 3.5 mg/kg intraperitoneal LPS (*Escherichia coli* 026:B6; Sigma-Aldrich, Co., St. Louis, MO, USA). On day 2, the 18 LPS-challenged mice were assigned to one of the following treatment groups (administered by gavage, 0.1 mL/animal/day): LPS group: PBS; LPS + fish oil (FO) group: FO (EPA 43%, DHA 20%, other *n*-3: 2%); and LPS + corn oil (CO): CO (62% *n*-6 LA). The FO and CO content accounted for ~6% of total energy intake. Food intake and body weight were measured every day and the study was terminated when the average body weight in the LPS group reached 20% of its value at baseline. At study termination, mice were sacrificed by intraperitoneal injection with ketamine (100 mg/kg) and xylazine (10 mg/kg), blood samples were taken and plasma was generated by centrifugation prior to storage at −80 °C for further analysis. Small intestine and gastrocnemius muscle tissue were dissected and immersed in liquid N_2_ before storage at −80 °C.

All animal experimental protocols were approved by the Institutional Animal Care and Animal Ethics Committee of Massachusetts General Hospital (Boston, MA, USA).

### 4.2. Systemic Inflammation Measurement

Concentrations of TNF-α, IL-1β, IL-6 and monocyte chemoattractant protein 1 (MCP-1), were determined in 15 μL of plasma and small intestine PBS homogenates using a multiplex immunoassay kit (Bio-Plex Pro™ Mouse Cytokine Assay, Bio-Rad, Waltham, MA, USA) and measured using Luminex^®^ technology (Bioplex^®^, Bio-Rad, Waltham, MA, USA). The extent of liver inflammation was determined by measurement of plasma alanine transaminase (ALT) and aspartate transaminase (AST) activity using Alanine Transaminase and Aspartate Transaminase Enzymatic Assay Kits (BIOO Scientific Corp., Austin, TX, USA) following the manufacturer’s protocols.

### 4.3. Gene Expression Measurement

Total RNA was isolated from small intestine and gastrocnemius muscle using Trizol reagent (Invitrogen, Carlsbad, CA, USA). cDNAs were synthesized from 1 μg of RNA using iScrip™ Reverse Transcription Supermix (Bio-Rad, Waltham, MA, USA) for RT-PCR. After cDNA synthesis, Q-PCR was performed in 20 μL of iTaqTM Universal SYBR^®^ Green Supermix (Bio-Rad, Waltham, MA, USA), using a fluorometric thermal cycler (Stratagene Mx3005P PCR Detection System, Agilent Technologies, Santa Clara, CA, USA). All primers were synthesized by Invitrogen (Carlsbad, CA, USA). All samples were processed in triplicate and normalized to β-actin. The expression levels were calculated using the ΔΔCt method after correcting for differences in PCR efficiencies, and values were expressed relative to those of the control group.

### 4.4. Western Blotting Analysis

Small intestine total protein extracts were prepared using RIPA buffer, and protein concentrations were measured using a BCA kit (Pierce, Rockford, IL, USA). Proteins (50 μg) were separated by electrophoresis on a 4%–12% SDS–PAGE gel and transferred to PVDF membranes (Invitrogen, Carlsbad, CA, USA). The primary antibodies were mouse anti-TLR4 and MyD88 (1:1000; GeneTex). The secondary antibodies were horseradish peroxidase (HRP)-conjugated ECL rabbit anti-mouse IgG (1:4000; Santa Cruz Biotechnology, Santa Cruz, CA, USA). Chemiluminescent detection was performed using the ECL method (Santa Cruz Biotechnology, Santa Cruz, CA, USA).

### 4.5. Statistical Analysis

All data are presented as the mean ± standard error of the mean. Statistical analysis was performed with ANOVA and Tukey’s test by using SPSS software version 17.0. A *p* < 0.05 was considered to be statistically significant.

## 5. Conclusions

In summary, our study demonstrates that FO intervention suppresses LPS-induced inflammation and weight loss, associated with down-regulation of pro-inflammatory targets of the TLR4 signaling pathway, and suggests that *n*-3 PUFA may serve as potential therapeutics for the management of inflammatory diseases.
